# The Impact of Seropositivity on Systemic Bone Loss in Rheumatoid Arthritis—A 3-Year Interim Analysis of a Longitudinal Observational Cohort Study

**DOI:** 10.3389/fmed.2022.885801

**Published:** 2022-06-09

**Authors:** Shan-Fu Yu, Jia-Feng Chen, Ying-Chou Chen, Yu-Wei Wang, Chung-Yuan Hsu, Han-Ming Lai, Hsiao-Ru He, Chi-Hua Ko, Wen-Chan Chiu, Tien-Tsai Cheng

**Affiliations:** ^1^Division of Rheumatology, Allergy, and Immunology, Department of Internal Medicine, Kaohsiung Chang Gung Memorial Hospital, Kaohsiung, Taiwan; ^2^Division of Rheumatology, Allergy, and Immunology, Department of Internal Medicine, Chiayi Chang Gung Memorial Hospital, Chiayi, Taiwan; ^3^School of Medicine, College of Medicine, Chang Gung University, Tayouan, Taiwan

**Keywords:** rheumatoid arthritis, bone mineral density, anti-cyclic citrullinated peptide antibodies, rheumatoid factor, fracture

## Abstract

**Objective:**

To explore the impact of seropositivity on systemic bone loss in rheumatoid arthritis (RA).

**Methods:**

We conducted an interim analysis of the RA registry. Patients were examined with dual-energy X-ray absorptiometry at baseline and again 3 years later. Participants were grouped into seropositive (SPRA) and seronegative (SNRA) based on the presence or absence of rheumatoid factor (RF) and/or anti-cyclic citrullinated peptide antibodies (ACPA). After matching (1:2) for age and sex, SNRA and SPRA patients were divided into groups A and B. Each matched group (A or B) was further subdivided according to the number of antibodies present (0, group I; 1, group II; 2, group III). Multiple ordinary least squares regression was used with the dependent variables to develop a model to predict bone mineral density (BMD) change.

**Results:**

A total of 477 participants who completed a 3-year observation period were included. After matching, 312 participants were enrolled (group A, 104; group B, 208). Three years later, group B had significant BMD reduction in the femoral neck (FN) (*p* < 0.001), total hip (TH) (*p* = 0.001), and first through fourth lumbar vertebrae (L1–4) (*p* = 0.006), while group A had bone loss only at FN (*p* = 0.002). Groups I, II, and III included 104, 52, and 156 participants, respectively. Compared to baseline, BMD decreased significantly at FN (*p* = 0.002) in group I, FN (*p* < 0.001) in group II, and FN (*p* < 0.001), TH (*p* = 0.002), and L1–4 (*p* = 0.016) in group III. In terms of regression-adjusted percent change in BMD, more significantly negative changes were found at all measured sites in group B (*p* < 0.001, all) and at TH and L1–4 within groups I-III (*p* for trend < 0.001 and < 0.001, respectively). Regardless of antibodies, anti-osteoporotic therapy can preserve bone density in RA patients.

**Conclusion:**

After 3 years, SPRA patients lost more bone density than SNRA patients. More attention should be paid to SPRA patients, especially those with double-positive antibodies, including a vigorous evaluation of BMD and fracture risk. Anti-osteoporotic therapy can prevent BMD loss irrespective of autoantibodies.

## Introduction

Rheumatoid arthritis (RA) is a chronic systemic disease that can lead to local bone erosion and generalized osteoporosis. Rheumatoid factor (RF) and anti-citrullinated protein antibodies (ACPA) are the two most notable autoantibodies commonly used in diagnosing or classifying RA and providing a variety of clinical and pathophysiological information (1). Patients positive for ACPA and/or RF may be labeled together as having “seropositive” RA (SPRA) and compose approximately 50–80% of the RA population (1). Several studies have indicated that SPRA patients experience greater disease severity in terms of disease activity, functional impairment, increased mortality over time (2, 3), and might show poor response to treatment compared to seronegative RA (SNRA) patients (4). Although both SPRA and SNRA fulfill the 1987 American College of Rheumatology (ACR) revised classification criteria (5) or the 2010 ACR/European League Against Rheumatism (EULAR) classification criteria (6), their clinical manifestations, courses, and prognostic features are distinctive.

It has been reported that the annual rate of bone loss in patients with active RA ranges between 5.5 and 10% (7). Meanwhile, RF and/or ACPA are associated with juxta-articular osteoporosis, erosions, and generalized bone loss (8, 9). Several studies have shown that ACPAs and RF synergize to promote RA-associated inflammation, disease activity, and clinical onset (10, 11) and can be associated with the bone erosion and structural damage seen in RA (12). The combination of ACPA and RF could predict the therapeutic responses to rituximab and abatacept (13). However, the clinical predictive value of each antibody (singly or in combination) on systemic bone loss has not been well studied.

This study aimed to explore long-term bone mineral density (BMD) changes in patients with SPRA and SNRA and investigate the association between antibody number and systemic bone loss in patients with RA.

## Materials and Methods

### Study Population and Design

Participants’ inclusion criteria and the methods used in this study were the same as those reported previously (14). Study participants enrolled in an RA registry at Kaohsiung Chang Gung Memorial Hospital beginning September 1st, 2014. We enrolled a total of 651 patients who satisfied the 1987 revised ACR criteria for RA (5) or the 2010 ACR/EULAR classification criteria for RA (6) in this study. Demographic characteristics such as age, sex, comorbidities, and body mass index were recorded.

We also recorded disease-specific information, such as the age at diagnosis, disease duration, disease activity measured by erythrocyte sedimentation rate (ESR), C-reactive protein, and the disease activity score-28 joint-ESR (DAS28-ESR), medications, including glucocorticoids (GC) and biological and targeted synthetic disease-modifying antirheumatic drugs (b/tsDMARDs), and the presence/absence of ACPA and RF. Lifestyle, evidence of previous fragility fracture (history or radiographic), and risk factors for fragility fracture on the Fracture Risk Assessment Tool (FRAX ^®^) were also recorded. The 10-year probabilities of major and hip fractures in each patient, calculated using the FRAX ^®^ with BMD (Taiwan version), were collected. Seropositivity was defined as any value > 15 IU/mL for RF and > 7 IU/mL for ACPA.

Each patient’s BMD was measured at enrollment and the 3-year follow-up using a dual-energy X-ray absorptiometry scanner (Delphi A; Hologic Corp., Waltham, MA, United States) for the femoral neck (FN), total hip (TH), and first through fourth lumbar vertebrae (L1–4). For postmenopausal women and men aged 50 years and older, osteoporosis was defined as a T-score of –2.5 or less at the FN based on the normal reference database for young white females (15, 16). We calculated the percentage change in BMD (ΔBMD%) for each participant as follows: [(second BMD – baseline BMD)/baseline BMD] × 100, comparing between assessments.

We defined a new incident fracture as any symptomatic non-traumatic fracture, including the forearm, hip, pelvis, and humerus, or an asymptomatic morphometric vertebral fracture. Morphometric fractures were assessed on lateral radiographs of the lumbar spine according to Genant et al.’s semiquantitative assessment of vertebral fractures (17). An independent radiologist assessed the evidence of morphometric vertebral compression fractures at enrollment and subsequently on an as-needed basis, with follow-up spinal radiographs during the 3-year observation period and at the end of the study. The local Institutional Review Board of Chang Gung Memorial Hospital approved the study (104-3530B, 201901054B0), which was performed according to the principles of the Declaration of Helsinki. Written informed consent was obtained from all study participants.

Patients who were positive for RF or ACPA were grouped into the SPRA group, while those who were negative were grouped as SNRA. The participants were sub-grouped into A and B after matching for age and sex. Finally, the matched group was sub-grouped according to the number of antibodies present (0, group I; 1, group II; 2, group III).

### Statistical Analysis

The data were checked for normality, which demonstrated that baseline characteristics had a skewed distribution; therefore, they were analyzed using non-parametric methods. An independent two-sample *t*-test and one-way analysis of variance (ANOVA) test were used to compare continuous variables with normal distribution and expressed as mean ± standard deviation (SD). We used the Mann-Whitney U and Kruskal-Wallis tests to compare continuous variables with skewed distribution; they were expressed as a median (interquartile range, IQR). The chi-square or Fisher’s exact test was used to assess the association between categorical variables.

The intra-and inter-group BMD changes from enrollment to 3 years later were compared with a linear mixed model. Trend analyses were performed using ANOVA for each category (number of antibodies). Multiple ordinary least squares regression was used with the dependent variables, controlling for disease duration, baseline DAS28-ESR, glucocorticoid, and b/tsDMARDs therapy. From this, we calculated the predicted value of the BMD changes. All statistical analyses were performed using IBM SPSS version 22 software (IBM Co., Armonk, NY, United States). A *p*-value of < 0.05 was considered significant in all analyses.

## Results

### Demographics and Clinical Characteristics of Seropositive RA and Seronegative RA Participants

**FIGURE 1:**
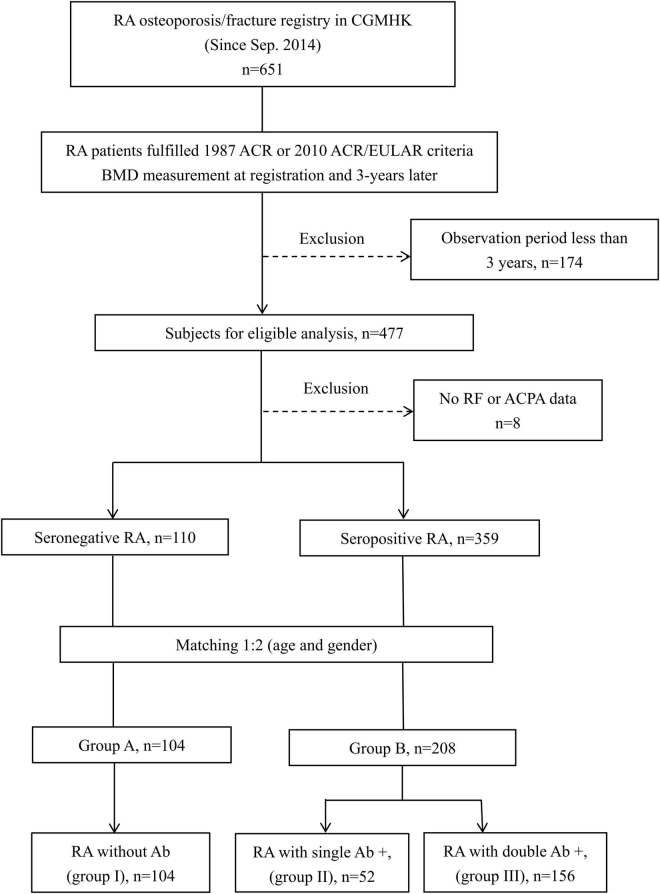
Disposition of participants and grouping.

The participants’ disposition is given in Figure 1. A total of 651 participants were registered for the RA osteoporosis/fracture study, which started on September 1st, 2014; 477 participants completed the 3-year observation period. To compare BMD changes in SNRA and SPRA, taking the mutual interference of age and sex into account, we controlled for these two factors. We obtained 312 matched participants, of whom 104 were allocated to group A and 208 to group B (Figure 1). The baseline DAS28-ESR and ESR were significantly higher in group B than in group A (*p* = 0.002 and *p* = 0.001, respectively) (Table 1, right column). The proportion of patients using GC and b/tsDMARDs in group B was higher than in group A (*p* = 0.017 and *p* = 0.004, respectively). No significant difference was found between the two groups in terms of the history of previous fractures (Table 1, right column). There were 134 patients receiving anti-osteoporotic therapy (AOT), of which 115 (85.8%) patients were treated with bisphosphonates, and 7 (5.2%) patients were treated with denosumab (Table 1, right column). The proportion of patients using bisphosphonates in group B was higher than in group A (*p* = 0.015). The mean treatment duration of bisphosphonates in group A and group B was 17.7 ± 30.3 and 15.5 ± 25.3 months, respectively (*p* = 0.697). The mean treatment duration of denosumab in group A and group B was 28.0 ± 33.0 and 22.5 ± 19.2 months, respectively (*p* = 0.790).

**TABLE 1:** Clinical characteristics of participants before and after matching.

	Before matching				After matching			
	Total (*n* = 469)	SNRA (*n* = 110)	SPRA (*n* = 359)	*p* ^a^	Total (*n* = 312)	Group A (*n* = 104)	Group B (*n* = 208)	*p* ^a^
Age, years	58 (14)	55 (14)	60 (13)	0.014	57 (14)	55 (14)	57 (14)	0.504
Female, n (%)	398 (84.9)	96 (87.3)	302 (84.1)	0.420	275 (88.1)	90 (86.5)	185 (88.9)	0.536
Postmenopause, n (%)	313 (78.6)	67 (69.8)	246 (81.5)	0.015	208 (75.6)	64 (71.1)	164 (77.8)	0.223
BMI, kg/m^2^	23.1 (5.1)	23.7 (5.0)	23 (5.1)	0.155	23.4 (5.2)	23.8 (5.2)	23.0 (5.4)	0.123
Comorbidities +, n (%)	278 (59.3)	63 (57.3)	215 (59.9)	0.625	190 (60.9)	63 (60.6)	127 (61.1)	0.935
**RA- related factors**								
Disease duration, years	13 (3)	12 (13)	13 (13)	0.870	12 (11)	12 (13)	12 (12)	0.765
Baseline DAS28-ESR	3.2 (1.7)	2.7 (1.6)	3.3 (1.6)	<0.001	3.0 (1.6)	2.7 (1.7)	3.3 (1.5)	0.002
Mean DAS28-ESR	3.0 (1.2)	2.8 (1.2)	3.0 (1.2)	0.017	2.9 (1.2)	2.9 (1.2)	3.0 (1.1)	0.088
HAQ-DI	0.25 (0.88)	0.13 (0.75)	0.25 (1.0)	0.092	0.25 (0.88)	0.13 (0.66)	0.25 (0.88)	0.295
ESR, mm/h	17 (21)	11.0 (13.0)	18.0 (24.0)	<0.001	16 (19)	11 (13.8)	17 (20.5)	0.001
**Fracture-related factors^b^ +**								
Parent fractured hip +, n (%)	36 (7.7)	10 (9.2)	26 (7.3)	0.522	26 (8.3)	10 (9.7)	16 (7.8)	0.571
Previous fracture +, n (%)	148 (31.6)	36 (32.7)	112 (31.2)	0.763	100 (32.1)	34 (32.7)	66 (31.7)	0.864
Alcohol +, n (%)	7 (1.5)	2 (1.8)	5 (1.4)	0.747	4 (1.3)	2 (1.9)	2 (1.0)	0.477
Smoking +, n (%)	31 (6.6)	4 (3.6)	27 (7.5)	0.190	15 (4.8)	4 (3.8)	11 (5.3)	0.575
Secondary osteoporosis +, n (%)	21 (4.5)	3 (2.7)	18 (5.0)	0.432	8 (2.6)	2 (1.9)	6 (2.9)	0.612
**BMD (g/m^2^)**								
FN	0.633 ± 0.120	0.648 ± 0.122	0.620 ± 0.117	0.014	0.641 ± 0.123	0.664 ± 0.114	0.627 ± 0.125	0.004
TH	0.789 ± 0.141	0.801 ± 0.140	0.775 ± 0.142	0.067	0.796 ± 0.143	0.818 ± 0.128	0.784 ± 0.147	0.015
L1–4	0.871 ± 0.170	0.885 ± 0.167	0.860 ± 0.168	0.129	0.874 ± 0.868	0.894 ± 0.167	0.863 ± 0.163	0.104
Osteoporosis^c^, n (%)	138 (29.4)	22 (16.2)	116 (24.9)	0.033	81 (26)	16 (15.4)	65 (31.3)	0.003
**FRAX score**								
Major	14 (18.9)	12 (13.5)	15 (19)	0.007	13 (18.1)	12 (13.9)	14 (20.2)	0.086
Hip	4.2 (8.4)	3.4 (6.2)	5.0 (9.5)	0.001	3.9 (8)	3.4 (6.1)	4.3 (9.5)	0.018
New incident fracture (3 years), n (%)	132 (28.1)	24 (26.1)	108 (34.8)	0.116	86 (27.6)	22 (25.3)	64 (36)	0.082
**Medications**								
GC +, n (%)	409 (87.2)	90 (81.8)	319 (88.9)	0.053	291 (93.3)	92 (88.5)	199 (95.7)	0.017
b/tsDMARDs^d^ + n (%)	84 (17.9)	12 (10.9)	72 (20.1)	0.031	79 (25.3)	16 (15.4)	63 (30.3)	0.004
AOT^e^ +, n (%)	163 (34.8)	30 (27.3)	133 (37)	0.060	134 (42.9)	42 (40.4)	92 (42.4)	0.518
BP +, n (%)	143 (87.7)	25 (83.37)	118 (88.7)	0.617	115 (85.8)	31 (73.8)	84 (91.3)	0.015
RANKLi +, n (%)	14 (8.6)	3 (10)	11 (8.3)	0.764	7 (5.2)	3 (7.1)	4 (4.3)	0.791

*Values are presented as mean ± standard deviation or median (interquartile range), unless otherwise mentioned. SNRA, seronegative rheumatoid arthritis; SPRA, seropositive rheumatoid arthritis; BMI, body mass index; DAS28-ESR, disease activity score-28 joint-erythrocyte sedimentation; HAQ-DI, health assessment questionnaire disability index; ESR, erythrocyte sedimentation rate; BMD, bone mineral density; FN, femoral neck; TH, total hip; L1–4, 1st–4th lumbar vertebra; FRAX, fracture risk assessment tool; GC, glucocorticoid; b/tsDMARDs, biologic/target synthetic disease modify anti-rheumatic drugs; AOT, Anti-osteoporotic therapy; BP, bisphosphonate; RANKLi, Receptor activator of nuclear factor-kB ligand inhibitor (denosumab).*

*+, presence.*

*^a^Comparison between seronegative and seropositive groups.*

*^b^Defined as in FRAX tool.*

*^c^T-score (femoral neck) ≤ −2.5.*

*^d^Including anti-TNFa (etanercept, adalimumab, golimumab, certolizumab), anti-IL6 receptor (tocilizumab), CTLA4 analog (abatacept), anti-CD 20 (rituximab), and JAK inhibitor, (tofacitinib).*

*^e^Including bisphosphonates, denosumab, teriparatide, and raloxifene during observation period.*

### Demographics and Clinical Characteristics of Group I–III Participants

Participants’ demographics and clinical characteristics are presented in Table 2. There were 104, 52, and 156 participants in groups I, II, and III, respectively. The baseline comorbidity (*p* = 0.010), DAS28-ESR (*p* = 0.006), ESR (*p* < 0.001), BMD at FN (*p* = 0.010), FRAX score (hip) (*p* = 0.049), proportion of osteoporosis (*p* = 0.011), proportion of b/tsDMARD use (*p* = 0.025), and proportion of bisphosphonates use (*p* = 0.041) were significantly different between groups. The mean treatment duration of bisphosphonates or denosumab was not significant in groups I–III.

**TABLE 2:** Clinical characteristics of participants grouped by presence of antibodies after matching.

	Group I	Group II	Group III	
	(*n* = 104)	(*n* = 52)	(*n* = 156)	*p* ^a^
Age, years	55 (14)	58 (13)	57 (14)	0.740
Female, n (%)	90 (86.5)	50 (96.2)	135 (86.5)	0.147
Postmenopause, n (%)	63 (71.1)	39 (78)	105 (77.8)	0.475
BMI, kg/m^2^	23.8 (5.2)	22.8 (5.3)	23 (5.4)	0.297
Comorbidities +, n (%)	63 (60.6)	41 (78.8)	86 (55.1)	0.010
**RA-related factors +**				
Disease duration, years	12 (13)	11.5 (19)	12 (10)	0.874
Baseline DAS28-ESR	2.7 (1.7)	3.0 (1.6)	3.3 (1.6)	0.006
Mean DAS28 at follow-up	2.8 (1.2)	2.9 (1.0)	3.0 (1.2)	0.233
HAQ-DI	0.13 (0.66)	0.25 (1.03)	0.25 (0.88)	0.442
ESR, mm/h	11 (13.8)	14 (20.3)	18 (22.8)	<0.001
**Fracture-related factors^b^ +**				
Parent fractured hip +, n (%)	10 (9.7)	2 (3.8)	14 (9.2)	0.420
Previous fracture +, n (%)	34 (32.7)	16 (30.8)	50 (32.1)	0.971
Alcohol +, n (%)	2 (1.9)	0 (0)	2 (1.3)	0.603
Smoking +, n (%)	4 (3.8)	0 (0)	11 (7.1)	0.103
Secondary osteoporosis +, n (%)	2 (1.9)	1 (1.9)	5 (3.2)	0.774
**Baseline BMD (g/m^2^)**				
FN	0.664 ± 0.114	0.635 ± 0.118	0.624 ± 0.127	0.010
TH	0.818 ± 0.128	0.782 ± 0.144	0.784 ± 0.148	0.053
L1–4	0.894 ± 0.167	0.868 ± 0.172	0.862 ± 0.160	0.254
**FRAX score**				
Major	12 (13.9)	13 (18.6)	14 (20.7)	0.214
Hip	3.4 (6.1)	4.4 (9.0)	4.3 (9.3)	0.049
New incident fracture (3 years), n (%)	22 (25.3)	14 (31.1)	50 (37.6)	0.159
Osteoporosis^c^ +, n (%)	16 (15.4)	16 (30.8)	49 (31.4)	0.011
**Medications**				
GC + , n (%)	92 (88.5)	49 (94.2)	150 (96.2)	0.050
b/tsDMARDs^d^ +, n (%)	10 (9.6)	11 (21.2)	35 (22.4)	0.025
AOT^e^ +, n (%)	42 (40.4)	25 (48.1)	67 (42.9)	0.658
BP +, n (%)	31 (73.8)	21 (84)	63 (94)	0.041
RANKLi +, n (%)	3 (7.1)	2 (8)	2 (3)	0.502

*Values are presented as mean ± standard deviation, and median (interquartile range), unless otherwise mentioned. BMI, body mass index; DAS28-ESR, disease activity score-28 joint-erythrocyte sedimentation; HAQ-DI, health assessment questionnaire disability index; ESR, erythrocyte sedimentation rate; BMD, bone mineral density; FN, femoral neck; TH, total hip; L1-4, 1st-4th lumbar vertebra; FRAX, fracture risk assessment tool; GC, glucocorticoid; b/tsDMARDs, biologic/target synthetic disease modify anti-rheumatic drugs; AOT, Anti-osteoporotic therapy; BP, bisphosphonate; RANKLi, Receptor activator of nuclear factor-kB ligand inhibitor (denosumab).*

*+, presence.*

*^a^Comparison among three groups.*

*^b^Defined as in FRAX tool.*

*^c^T-score (femoral neck) ≤ −2.5.*

*^d^Including anti-TNFa (etanercept, adalimumab, golimumab, certolizumab), anti-IL6 receptor (tocilizumab), CTLA4 analog (abatacept), anti-CD 20 (rituximab), and JAK inhibitor (tofacitinib).*

*^e^Including bisphosphonates, denosumab, teriparatide, and raloxifene during observation period.*

### Comparison of Bone Mineral Density Changes With Baseline in Groups A and B

**FIGURE 2:**
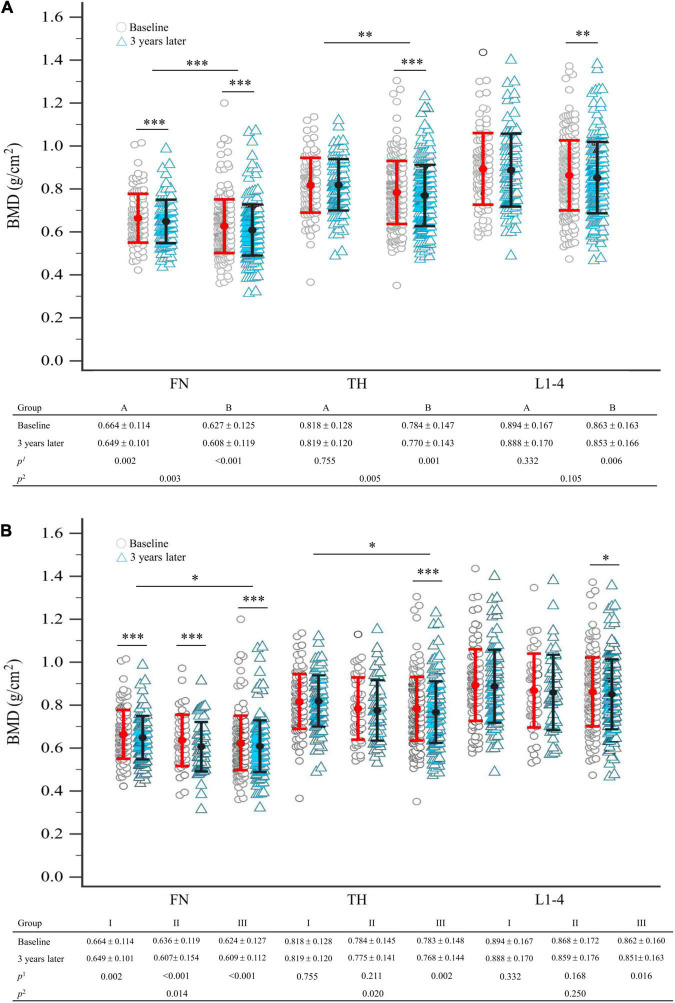
**(A)** Comparison of BMD between baseline and 3 years later between seronegative (group A) and seropositive (group B) rheumatoid arthritis, after matching; **(B)** comparison of BMD between baseline and 3 years later in total groups stratified by the number of antibodies presented, after matching. Statistical analysis using the linear mixed model; *p*^1^-value refers to intra-group comparison; *p*^2^-value refers to inter-group comparison. The error bar represents the standard deviation for the means. Each symbol represents a single data point. BMD, bone mineral density; FN, femoral neck; L1–4, first through fourth lumbar vertebra; TH, total hip. *p < 0.05; ^**^p < 0.01; ^***^p < 0.005.

Group B had a significantly higher proportion of patients with osteoporosis (*p* = 0.003) and a higher 10-year probability of hip fracture (*p* = 0.018) but had lower BMD at FN and TH (*p* = 0.004, *p* = 0.005) at enrollment, compared to group A (Table 1, right column). Three years later, the BMDs at FN, TH, and L1–4 were significantly decreased from baseline in group B (*p* < 0.001, *p* = 0.001, *p* = 0.006), respectively (Figure 2A). In group A, a significant decrease was found only in FN (*p* = 0.002) (Figure 2A). Intergroup differences over the three intervening years were significant at the FN and TH (*p* = 0.003 and *p* = 0.005, respectively), but were insignificant at L1–4 (*p* = 0.105) (Figure 2A). The difference in the rate of new incident fractures was not obvious between groups A and B over time (Table 1, right column). The most common types of new incident fractures were vertebral fractures (*n* = 61), followed by non-hip, non-vertebral fractures (*n* = 24), hip fractures (*n* = 8), and wrist fractures (*n* = 3) in all groups.

### Comparison of Bone Mineral Density Changes With Baseline in Groups I, II, and III

After 3 years, compared to baseline, significant BMD reductions were seen in group III participants at FN, TH, and L1–4 (*p* < 0.001, *p* = 0.002, *p* = 0.016, respectively). However, we demonstrated that groups I and II had significant BMD reductions only at FN (*p* = 0.002, *p* < 0.001, respectively) and not at TH or L1–4 (Figure 2B). Intergroup BMD differences over 3 years were significant at FN and TH (*p* = 0.014, *p* = 0.020), but not at L1–4 (*p* = 0.250) (Figure 2B).

### Differences in Percent Change of Bone Mineral Density Among Groups After Matching Age and Sex

**FIGURE 3:**
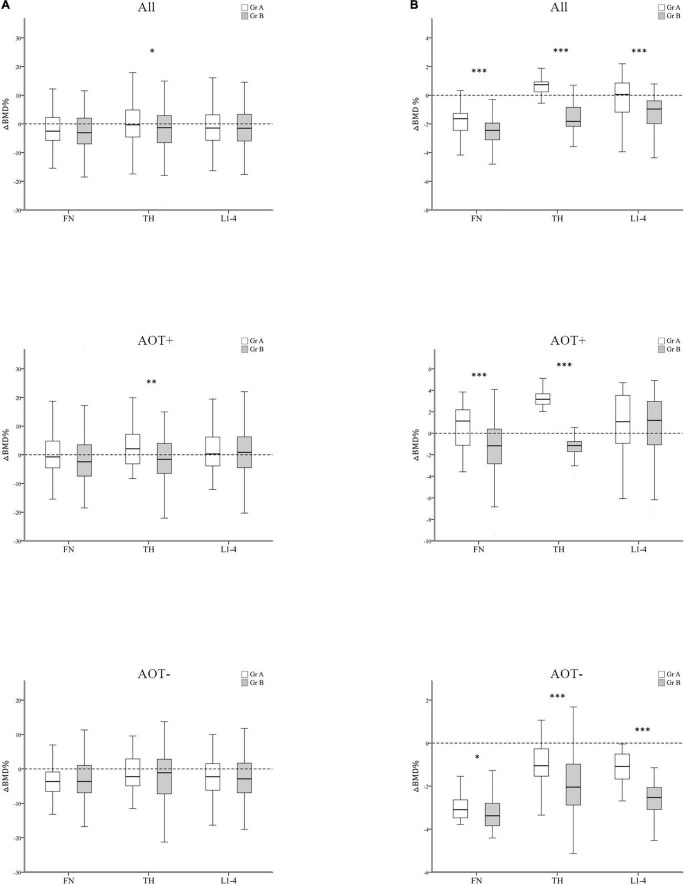
Regression-adjusted percentage change in BMD (ΔBMD%) between group A and group B, after matching in all participants and participants with or without anti-osteoporotic therapy (AOT). Unadjusted **(A)** and regression-adjusted **(B)** percentage of change in BMD at the femoral neck (FN), total hip (TH), and first through fourth lumbar vertebra (L1–4) after 3 years in group A and group B, combined with AOT presence. Box-and-whisker plots showed the median, interquartile range, and extreme values. *p < 0.05; ^**^p < 0.01; ^***^p < 0.005.

**FIGURE 4:**
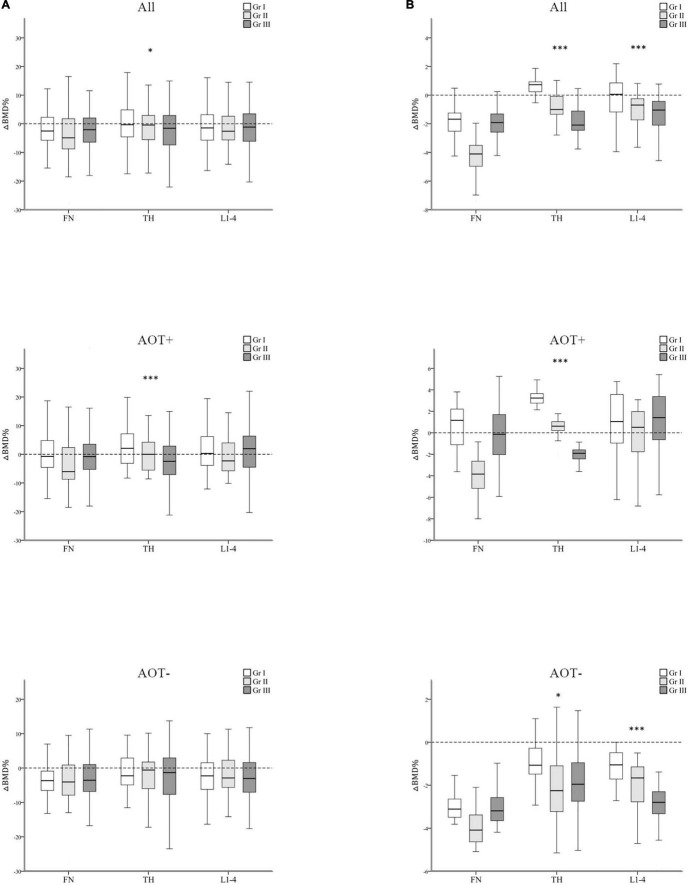
Regression-adjusted percentage change in BMD (ΔBMD%) from baseline in groups I–III, after matching in all participants and participants with or without anti-osteoporotic therapy (AOT). Unadjusted **(A)** and regression-adjusted **(B)** percentage of change in BMD at the femoral neck (FN), total hip (TH), and first through fourth lumbar vertebra (L1–4) after 3 years in groups I–III, combined with AOT presence. Box-and-whisker plots showed the median, interquartile range, and extreme values. *p for trend < 0.05; ^***^p for trend < 0.005.

After 3 years, percent changes in BMD (ΔBMD%) at FN and L1–4 were not significantly different among groups except at TH (*p* = 0.044) after matching in group A and group B participants (Table 3 and Figure 3A). ΔBMD% at FN and L1–4 were not obviously different among group A and group B participants with AOT (*p* = 0.121, 0.970, respectively) or without (*p* = 0.908, 0.650, respectively) (Table 3). However, ΔBMD% at TH in group B were significantly different from group A participants with AOT (*p* = 0.009). In all participant groups, patients with an increasing number of autoantibodies were associated with a trend toward more negative ΔBMD% at TH (*p* for trend 0.021, Table 4 and Figure 4A). Among participants with AOT, patients with a higher number of antibodies had more negative ΔBMD% at TH compared with patients with few autoantibodies (*p* for trend 0.002, Table 4 and Figure 4A).

**TABLE 3:** Regression-adjusted percentage change in BMD from baseline in each group, after matching.

Group	△BMD%^a^					
	FN		TH		L1–4	
	Unadjusted	Adjusted^c^	Unadjusted	Adjusted^c^	Unadjusted	Adjusted^c^
**Total**						
Gr A	−2.52 (8.37)	−1.64 (1.19)	−0.27 (9.43)	0.73 (0.70)	−1.45 (9.35)	0.05 (2.05)
Gr B	−3.08 (9.04)	−2.45 (1.17)	−1.36 (9.61)	−1.82 (1.33)	−1.50 (9.44)	−0.96 (1.59)
*p*-value^b^	0.362	<0.001	0.044	<0.001	0.807	<0.001
**AOT +**						
Gr A	−0.74 (10.66)	1.14 (3.34)	2.10 (10.04)	3.17 (0.97)	0.30 (10.33)	1.08 (4.55)
Gr B	−2.21 (10.93)	−1.09 (3.28)	−2.43 (10.71)	−1.15 (0.98)	0.85 (10.9)	1.21 (4.05)
*p*-value^b^	0.121	<0.001	0.009	<0.001	0.970	0.494
**AOT–**						
Gr A	−3.64 (5.89)	−3.10 (0.87)	−2.22 (7.98)	−1.03 (1.27)	−2.27 (8.30)	−1.09 (1.17)
Gr B	−3.65 (7.98)	−3.38 (1.06)	−1.12 (10.45)	−2.05 (2.02)	−2.90 (8.72)	−2.53 (1.06)
*p*-value^b^	0.908	0.032	0.705	0.001	0.650	<0.001

*FN, femoral neck; L1–4, lumbar vertebrae 1–4; TH, total hip; BMD, bone mineral density; Gr, group; AOT, anti-osteoporotic therapy.*

*Data are presented as median (interquartile range).*

*+, presence; −, absence.*

*^a^△BMD%: [(BMD 3 years later - BMD at baseline)/BMD at baseline] × 100%.*

*^b^Comparison of △BMD% among groups at each site.*

*^c^Predicted change in BMD was calculated by multiple regression analysis after adjusting disease duration, baseline DAS28-ESR, glucocorticoid, b/tsDMARDs therapy.*

**TABLE 4:** Regression-adjusted percentage change in BMD from baseline in each group, after matching.

Group	△BMD%^a^					
	FN		TH		L1–4	
	Unadjusted	Adjusted^c^	Unadjusted	Adjusted^c^	Unadjusted	Adjusted^c^
**Total**						
Gr I	−2.53 (8.50)	−1.68 (1.26)	−0.25 (9.56)	0.73 (0.69)	−1.45 (9.17)	0.56 (2.05)
Gr II	−4.81 (11.32)	−4.10 (1.57)	−0.57 (7.92)	−1.00 (1.38)	−2.60 (8.35)	−0.69 (1.54)
Gr III	−2.07 (8.81)	−1.96 (1.29)	−2.04 (10.59)	−2.10 (1.35)	−1.28 (9.62)	−1.04 (1.67)
*p* for trend^b^	0.960	0.739	0.021	<0.001	0.434	<0.001
**AOT +**						
Gr I	−0.74 (10.66)	1.17 (3.24)	−2.10 (10.04)	3.24 (0.87)	0.30 (10.33)	1.05 (4.60)
Gr II	−6.72 (12.85)	−3.68 (2.47)	−0.16 (10.25)	0.66 (1.05)	−2.31 (11.05)	0.51 (4.06)
Gr III	−0.81 (9.07)	−0.95 (3.73)	−2.77 (10.98)	−1.90 (0.87)	1.96 (11.04)	1.41 (4.08)
*p* for trend^b^	0.744	0.260	0.002	<0.001	0.909	0.867
**AOT −**						
Gr I	−3.64 (5.89)	−3.12 (0.88)	−2.22 (7.89)	−1.04 (1.26)	−2.27 (8.30)	−1.05 (1.25)
Gr II	−3.58 (10.60)	−4.00 (1.41)	−0.87 (8.87)	−2.39 (2.72)	−2.90 (7.95)	−1.66 (1.75)
Gr III	−3.65 (7.88)	−3.20 (1.09)	−1.20 (10.69)	−1.94 (1.87)	−3.04 (8.69)	−2.80 (1.04)
*p* for trend^b^	0.849	0.299	0.671	0.027	0.194	<0.001

*FN, femoral neck; L1–4, lumbar vertebrae 1–4; TH, total hip; BMD, bone mineral density; Gr, group; AOT, anti-osteoporotic therapy.*

*Data are presented as median (interquartile range).*

*+, presence; −, absence.*

*^a^△BMD%: [(BMD 3 years later - BMD at baseline)/BMD at baseline] × 100%.*

*^b^a trend of ΔBMD% in Gr I-III.*

*^c^Predicted change in BMD was calculated by multiple regression analysis after adjusting disease duration, baseline DAS28-ESR, glucocorticoid, b/tsDMARDs therapy.*

Next, we calculated the predicted BMD change by multiple regression analysis after adjusting for disease duration, baseline DAS28-ESR, glucocorticoid, and b/tsDMARDs therapy (Tables 3, 4 and Figures 3, 4). In all participant groups, regression-adjusted ΔBMD% at all measured sites in group B were significantly different from those of group A (all *p* < 0.001) (Table 3 and Figure 3B). Regression-adjusted ΔBMD% at FN and TH in group B were significantly different from group A participants with AOT (*p* < 0.001, < 0.001, respectively). Among participants without AOT, regression-adjusted ΔBMD% at all measured sites in group B were significantly different from those of group A (all *p* < 0.005).

In all participant groups, patients with an increasing number of autoantibodies were associated with a trend toward more negative regression-adjusted ΔBMD% at TH and L1-4 (*p* for trend < 0.001, < 0.001, respectively, Table 4 and Figure 4B). Among participants with AOT, patients with a higher number of antibodies had more negative adjusted ΔBMD% at TH compared with patients with few autoantibodies (*p* for trend < 0.001). Among participants without AOT, patients with a higher number of antibodies were more negative adjusted ΔBMD% at TH and L1–4 compared with patients with few autoantibodies (*p* for trend 0.027, < 0.001, respectively).

## Discussion

Our study provides 3-year follow-up results on the impact of seropositivity and the number of autoantibodies on BMD changes in patients with RA. Compared to baseline, SPRA patients experienced a significant decrease in BMD at three measured sites but not in SNRA at TH and L1–4. Regardless of AOT, autoantibodies present could increase the progression of bone loss at all sites. In patients without AOT, SPRA participants had the most obvious bone loss at all sites. Furthermore, we found that patients with higher numbers of antibodies had more systemic bone loss at TH and L1–4, irrespective of taking AOT therapy or not.

It has been well documented that RA patients have a higher risk of osteoporosis and fragility fractures than the general population (18). RA-associated bone loss is not only related to traditional risk factors of osteoporosis (e.g., aging, female) but also to factors related to the disease itself (e.g., disease activity and duration, GC use, and functional disability) (19, 20). Biologics for patients with RA had a protective effect on bone loss. This beneficial effect was also observed in patients who did not exhibit a clinical response (21). However, it is still controversial whether seropositivity affects osteoporosis and BMD changes in RA. Some studies have reported that SNRA patients have a greater chance of developing osteoporosis and lower BMD than SPRA patients (22, 23), but other investigations revealed opposite results (17, 24–26). The aforementioned studies were subjected to a cross-sectional study with a small sample size and no confounding factor adjustment. In terms of the influential factors screened, we conducted a 3-year, longitudinal, observational study with adequate sample size and adjusted the analysis, factoring in confounders. Our study’s results suggest that seropositivity has a detrimental effect on systemic bone loss in RA patients.

Recent investigations have suggested that not only do SPRA patients have distinct genomic backgrounds (27), clinical presentations (28), and treatment responses (29) from SNRA patients, but they also have higher mortality (3). It raises the possibility that SPRA and SNRA are two distinctive disease entities that mediate the different patterns of systemic bone loss seen in these two subtypes of RA (17). Current investigations revealed that RA-related factors and treatment, including baseline DAS28-ESR, ESR, and rate of GC and biologics use, were significantly higher in SPRA than SNRA, which further suggests that these subtypes of RA are different disease entities. RA disease activity (19, 30) and GC use (21, 31) are two of the determinants of RA-related systemic bone loss, suggesting that higher disease activity and greater GC usage are important determinants of greater systemic bone loss and a higher proportion of osteoporosis in SPRA than SNRA in our cohort.

In addition to the indirect effect of different disease patterns on bone loss between SPRA and SNRA, the direct effect of seropositivity on bone loss has been demonstrated. Harre et al. revealed an association between autoantibodies against citrullinated vimentin and serum markers for osteoclast-mediated bone resorption in RA patients (32). RA-associated autoantibodies have recently been found to directly induce differentiation and activation of osteoclasts, which might partly mediate systemic bone loss in RA (33–35). The additive effect of ACPA and RF on the production of the pro-inflammatory cytokine TNF-α, which is among the most potent cytokines to stimulate osteoclastogenesis, has been noted (10, 36, 37). This may explain the antibody-dependent enhancement of systemic bone loss and the concurrence of ACPA and RF in RA patients having the most detrimental effect on BMD in groups I–III. We noticed that more negative regression-adjusted ΔBMD% at TH and L1–4, irrespective of taking AOT therapy, were considered expected factors of the concurrence of RF and ACPA.

Interestingly, we found that not only is substantial bone loss consistent compared to baseline at three measured sites either in SPRA or RA patients with more autoantibodies, but regression-adjusted ΔBMD% over time is also more negative at all sites, especially at TH. This is consistent with previous studies’ findings suggesting that RA patients experienced more bone loss at the hip than the spine (38–40). Orsolini et al. demonstrated that the independent role of ACPA has a negative titer-dependent effect on systemic bone loss, in particular at cortical sites such as the hip (41).

In contrast, results from Bugatti’s analysis indicate that, despite inflammation suppression, spine BMD is sensitive to systemic bone loss in ACPA-positive early RA patients during the first 2 years after treatment onset (42). A Swedish ACPA-positive sub-cohort study demonstrated a trend in reduced BMD at the spine and hip during the first 2 years of treatment (43). Based on the past studies’ findings (38–43), we hypothesized that ACPA and/or RF’s osteoclastogenesis effect on cortical and cancellous bones are different, and the longitudinal assessments of BMD variations in RA are complicated. Further work will hopefully clarify this controversial concern.

The FRAX ^®^ was launched in 2008 to allow health providers to estimate individual 10-year probabilities of fragility fractures (44); it is a free, reliable, and validated tool that is used globally. The current investigation found a significantly higher FRAX score (hip) in SPRA participants regardless of the presence of a single or double-positive antibody. Despite higher FRAX score and greater bone loss in SPRA patients, no significant difference was observed in previous fractures and new incident fractures over time between SPRA and SNRA, and in groups I–III. This suggests that more RA patients and a longer period of observation are needed to determine whether RA seropositivity also has a detrimental effect on incident fracture.

Osteoporosis is characterized by low bone mass, increased bone loss, and micro-architectural disruption, resulting in bone fragility. Based on epidemiological data, an operational definition of osteoporosis was defined as BMD lower than −2.5 SD below the peak bone mass in young adult white women (15, 16). This reference standard can be used to compare the results between studies and assess the accuracy of novel diagnostic tools. In clinical application, the focus lies more on bone quantity which refers to bone mass or density, whereas new technologies target the assessment of bone strength and bone quality information. The current study demonstrated that ACPA and RF were associated with BMD change. Whether these autoantibodies can affect bone strength or bone quality in RA patients warrants further investigation.

As the current study is a real-world investigation, we did not exclude participants who received AOT during the observation period to explore the interaction between autoantibodies and AOT in terms of bone protective effects. Among participants with AOT, there was significant bone loss at FN and TH in SPRA and at TH in patients with higher numbers of antibodies. Respective of antibodies, AOT had a better protective effect against loss of bone density in all sites, especially in the spine. This result echoes Pazianas et al.’s finding that bisphosphonates have a more pronounced effect on trabecular bone than on cortical bone (45).

The strengths of our study are as follows. As a real-world investigation, we thoroughly documented the reported clinical variables that may be associated with osteoporosis or fracture in RA patients to avoid the possible factors related to BMD changes that were not observed in previous investigations. Our initial investigation revealed a significant difference in the distribution of age between SNRA and SPRA groups. As age and sex are two of the most important determinants of BMD, we performed a 1:2 matching for age and sex to exclude the confounding effects of age and sex, which had not been done in previous investigations. Moreover, to adjust for confounding factors of bone loss, we used multiple regression analysis to establish a model for predicting BMD changes. We also explored the additive effects of ACPA and RF on systemic bone loss in RA, which has not been investigated before. Because of the 3-year observation period, we were able to investigate the link between autoantibodies’ presence and BMD at baseline and determine the impact of these autoantibodies on BMD changes over time.

This study had several limitations. The monocentric observational design used to diagnose RA patients allowed us to conclude our data about associations but not about causal relationships. Our patients did not represent an inception cohort; we presented the data at the time of study initiation, not at the time of RA diagnosis. Some of the measurements, including 25(OH) vitamin D and parathyroid hormone, were performed at baseline only (data not shown); serial measurements might be more suitable for exploring these associations. We did not check bone markers throughout the study, which hinders our understanding of the cohort’s pathogenesis of antibodies on systemic bone loss. Finally, RA-associated antibodies have been associated with the occurrence and progression of bone erosions and periarticular bone loss as well as a decreased BMD. Systemic bone loss might increase susceptibility to focal bone erosions in RA patients. We did not collect the erosion scores such as the vdH Sharp score or Larsen score initially and at the 3-year follow-up. Further study is needed to clarify the relationship between autoantibodies to bone density and focal bone damage.

## Conclusion

Compared to SNRA patients, SPRA patients had a higher prevalence of osteoporosis, consistent bone loss at all sites, and a higher 10-year probability of hip fracture. We also found that more autoantibodies in RA were associated with more detrimental effects on BMD. We, therefore, suggest that more attention should be paid to osteoporosis and systemic bone loss in SPRA patients, especially double antibody-positive patients. AOT had a better protective effect against BMD loss irrespective of autoantibodies. As for fragility fractures, further investigations are needed to explore the association between RA seropositivity and future fractures in the long term.

## Data Availability Statement

The original contributions presented in this study are included in the article/supplementary material, further inquiries can be directed to the corresponding author/s.

## Ethics Statement

The studies involving human participants were reviewed and approved by the Local Institutional Review Board of Chang Gung Memorial Hospital approved the study (104-3530B, 201901054B0). The patients/participants provided their written informed consent to participate in this study.

## Author Contributions

S-FY, J-FC, and T-TC were responsible for analysis and data interpretation. S-FY and T-TC were responsible for scientific writing. All authors read and approved the final manuscript and were involved with study conceptualization, design, and reporting.

## Conflict of Interest

The authors declare that the research was conducted in the absence of any commercial or financial relationships that could be construed as a potential conflict of interest.

## Publisher’s Note

All claims expressed in this article are solely those of the authors and do not necessarily represent those of their affiliated organizations, or those of the publisher, the editors and the reviewers. Any product that may be evaluated in this article, or claim that may be made by its manufacturer, is not guaranteed or endorsed by the publisher.
